# Case Report: Double Pectus Up in severe pectus excavatum, the new frontier of modified taulinoplasty

**DOI:** 10.3389/fped.2024.1399202

**Published:** 2024-05-17

**Authors:** Simone Frediani, Letizia Corbi, Valerio Pardi, Ivan Pietro Aloi, Arianna Bertocchini, Antonella Accinni, Simone Reali, Paolo Maria Salvatore Schingo, Alessandro Inserra

**Affiliations:** ^1^ General and Thoracic Pediatric Surgery Unit, Bambino Gesù Children’s Hospital, IRCCS, Rome, Italy; ^2^ Department of Anesthesia and Critical Care, Bambino Gesù Children’s Hospital, IRCCS, Rome, Italy; ^3^ Department of Imaging, Bambino Gesù Children’s Hospital, IRCCS, Rome, Italy

**Keywords:** taulinoplasty, Pectus Up, pectus excavatum, thoracic wall malformation, children

## Abstract

**Introduction:**

Pectus excavatum, also known as “sunken chest” or “funnel chest,” is a congenital condition where the sternum caves inward, creating a noticeable depression in the chest. This deformity can range from mild to severe cases, and can affect appearance and lung and heart function. Treatment options vary depending on the severity of the condition and associated symptoms. A case study was conducted on three patients suffering from severe forms of pectus excavatum using modified taulinoplasty with two Pectus Up bars.

**Case description:**

The patients were males, with an age of 15 years. Preoperatively, they underwent spirometry, an echocardiogram, and allergy tests. The procedure involved inserting two Pectus Up bars into the chest wall at the major sternal defect, allowing the implant to remain completely invisible. The procedure involved placing the sternal plate at the deepest point and anchored to the sternum with five self-tapping screws. The chest plate was then fixed to the bar using two screws.

**Discussion:**

The use of Pectus Up was first reported in 2016 and has been a subject of scientific discussion and research. The double Pectus Up technique offers improved correction, increased stability, and reduced complications. However, it also presents challenges such as increased technical complexity and potential for prolonged operative times. Patient outcomes showed promising results in terms of short-term correction and long-term stability. The use of a double bar technique in the modified Taulinoplasty procedure is a key area of ongoing clinical research and innovation in pectus excavatum repair. Further studies will be needed, including more institutions that use this technique to validate our initial experience.

## Introduction

Pectus excavatum, commonly referred to as “sunken chest” or “funnel chest,” is a congenital condition where the sternum caves inward, creating a noticeable depression in the chest. This deformity can vary in severity, ranging from mild to severe cases. While some individuals may only have a slight indentation, others may experience significant compression of the chest wall, which can affect both appearance and, in severe cases, lung and heart function. Pectus excavatum typically becomes noticeable during adolescence when growth spurts occur, as the condition may worsen during periods of rapid skeletal growth. It's sometimes associated with certain connective tissue disorders or syndromes, but most cases occur in otherwise healthy individuals without any underlying conditions (1). Treatment options vary depending on the severity of the condition and any associated symptoms. Mild cases may not require any treatment beyond monitoring, while more severe cases may benefit from surgical intervention. Several surgical procedures can be used to correct pectus excavatum, with the choice depending on the severity of the condition, the patient's age, overall health, and the personal experience of the surgeon. Here we report our case study on the use of two Pectus Up bars on adolescents suffering from severe forms of pectus excavatum. To our knowledge, these are the first cases reported in the literature using this technique.

## Cases description

**Figure 1:**
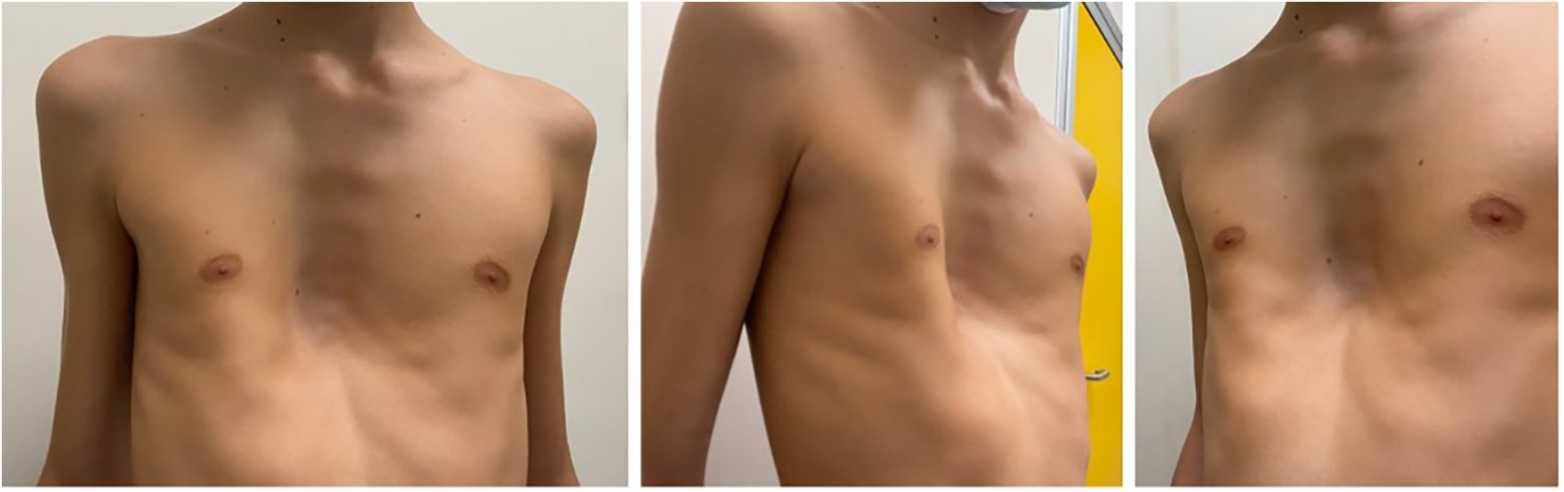
Pre-operative photo.

**Figure 2:**
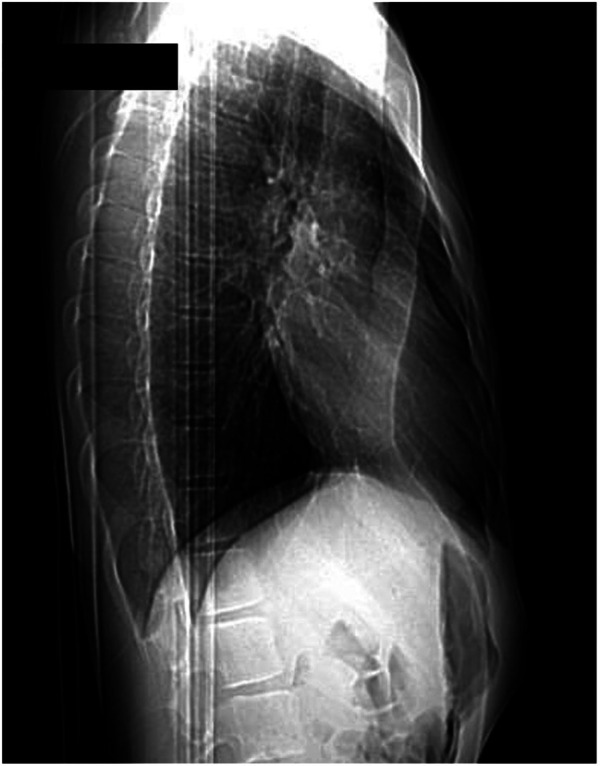
Pre-operative chest x-ray to measure the thickness of the sternum.

**Figure 3:**
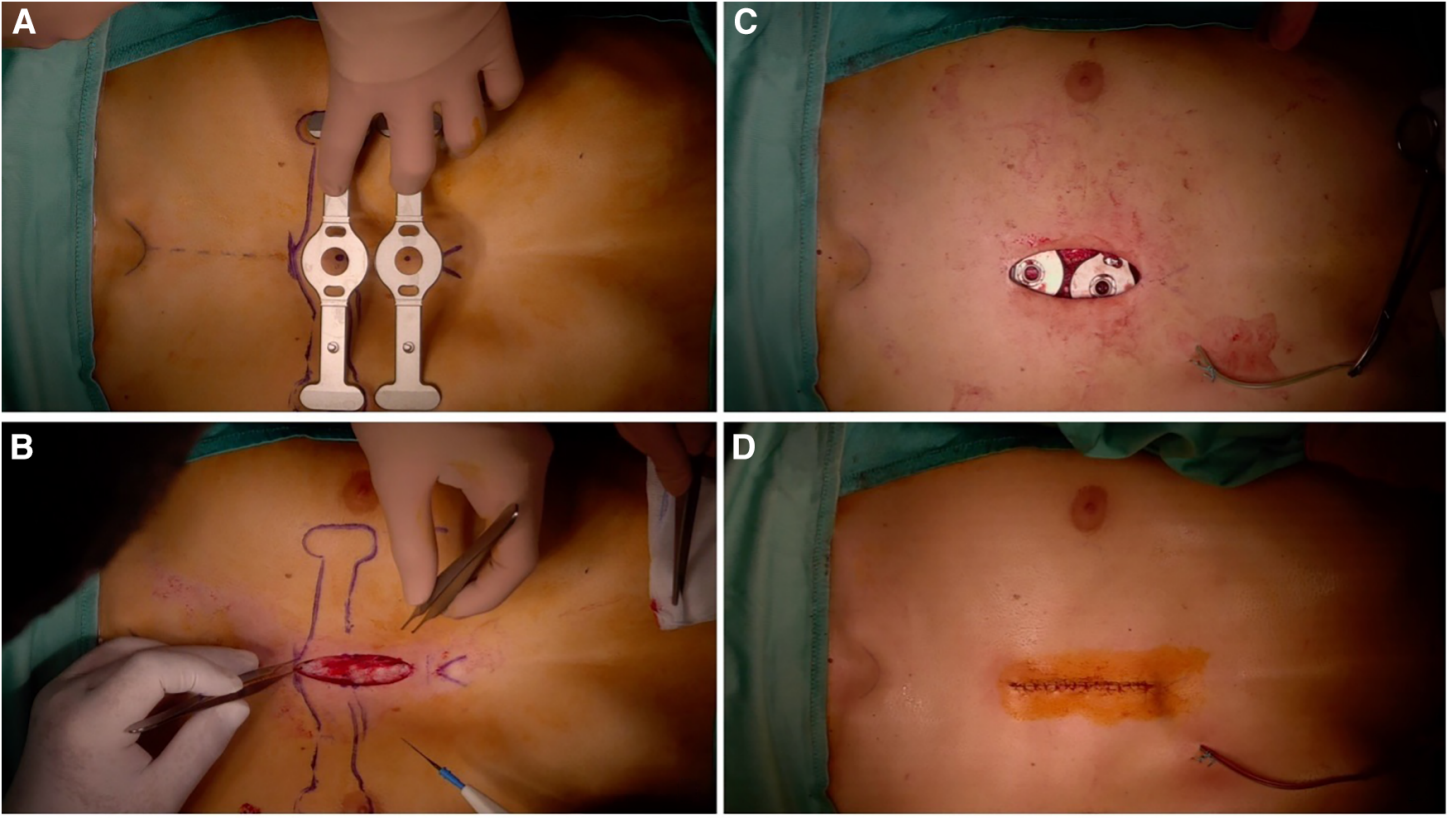
Intraoperative photo.

**Figure 4:**
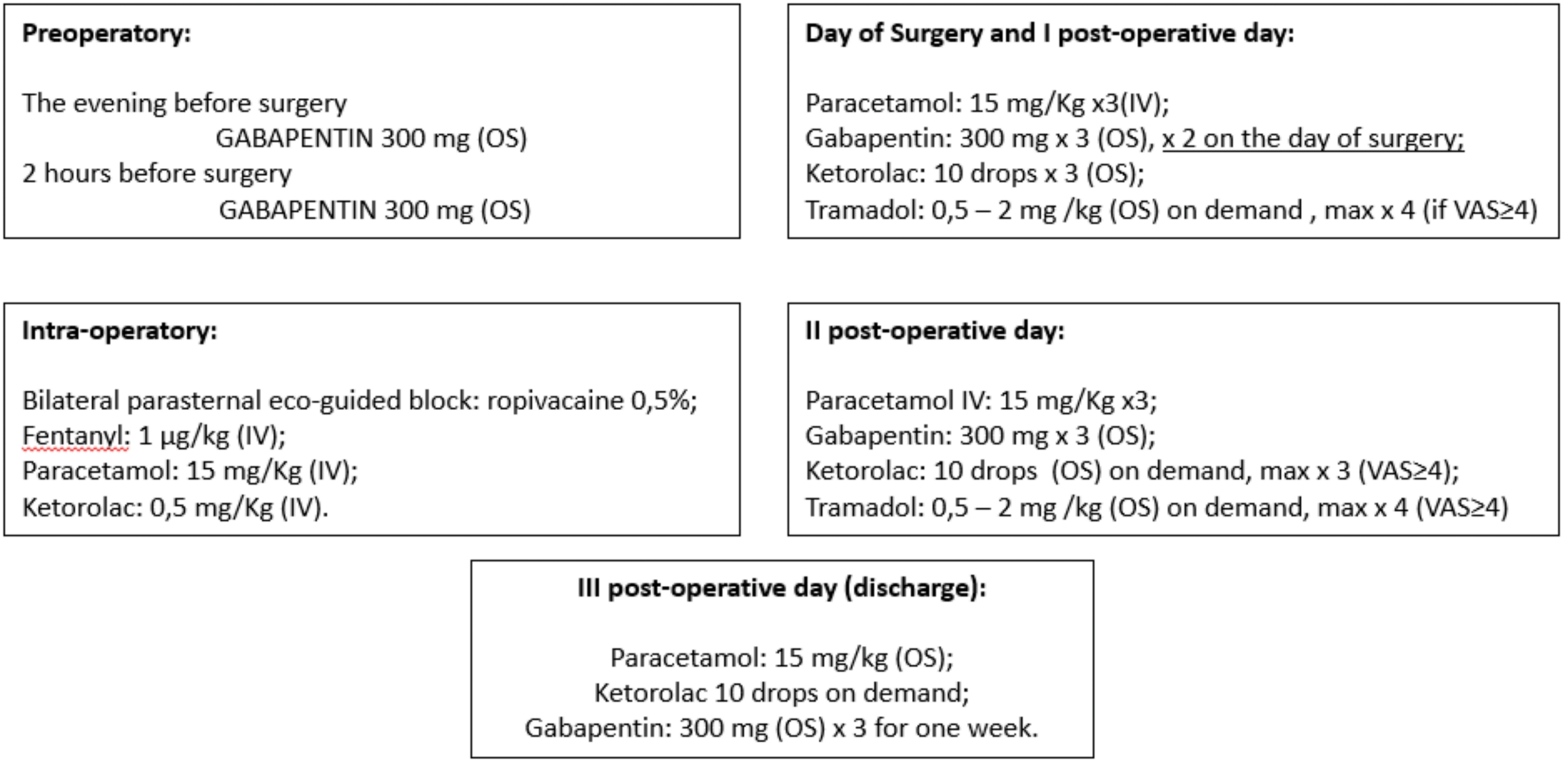
Pain management protocol for taulinoplasty (OS, oral somministration; VAS, visual analogue scale for pain).

We report our case series of three patients who underwent surgery for severe pectus excavatum using modified taulinoplasty with two Pectus Up (Figure 1). The patients are all males, with an age of 15 years. All patients preoperatively underwent spirometry, an echocardiogram, and allergy tests (nickel, chromium, and cobalt), all of which were negative. Before surgery, the patients underwent a chest x-ray performed latero-laterally at a fixed distance of 1.5 meters to measure the thickness of the sternum (Figure 2). Furthermore, the length of the pectus-up bar was measured as the distance between the two nipples. Insertion of the two Pectus Up bars was performed similar to the procedure of single-bar insertion. Briefly, the procedure was performed as follows: Patients were placed in the supine position, with both arms hanging freely from the overhead crossbar on the operating table. The entire anterior chest and both groins were prepared and draped (Figure 3A). A skin incision was performed vertically on the chest wall at the major sternal defect (Figure 3B). The implant is inserted into the compartment under the pectoral muscle after its medial disconnection. The implant is inserted in a deep position to allow it to remain completely invisible. A new bilateral sac was created under the pectoral muscles to house the device. In the case of double bar insertion, the devices penetrated through the target area, and the first was positioned over the second. The sternal plate is then positioned at the deepest point and anchored to the sternum with five self-tapping screws (Figure 3C). Subsequently, the chest plate is placed on the bag, and the traction system coupled with the double screw is mounted. By tightening the traction system, the defect is progressively reduced. The plate is then fixed to the bar using two screws. The incision is closed in layers after placing a drain under suction for 48 h (Figure 3D). Our protocol for post-operative pain does not differ from what is expected for patients undergoing surgery with single bar positioning (Figure 4). The patients were discharged on the third post-operative day. Subsequent checks revealed no complications.

## Discussion

**Figure 5:**
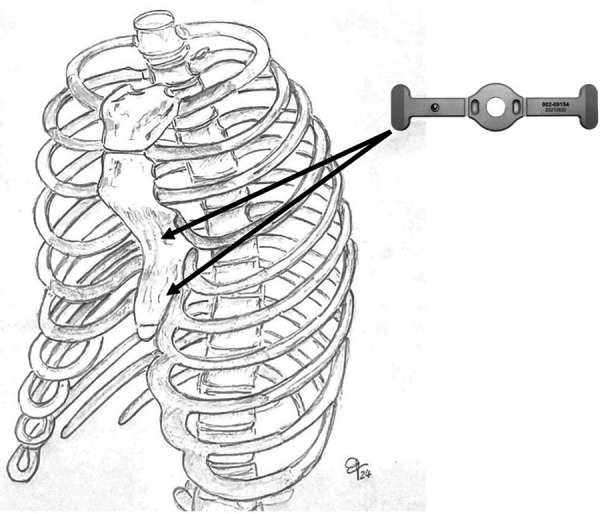
In cases of the broad or long depressions, two bars are inserted at superior and inferior levels parallel to each other.

Among congenital malformations of the chest wall, over 90% of cases concern the pectus excavatum, one of the most widespread congenital defects of the chest wall. A dip in the sternum and surrounding costal cartilages is its defining feature. Usually, it affects the bottom portion of the sternum. Depending on the degree of deformity, the compressed anterior chest wall causes impairment in cardiopulmonary function by compressing the intrathoracic organs, such as the heart and lungs. Additionally, it lessens psychosocial stress, which might restrict social interactions. The use of Pectus Up was first reported in 2016 (2). While the use of a double bar technique in the Nuss procedure for correcting severe pectus excavatum has been a subject of scientific discussion and research, to our knowledge, the use of a double Pectus Up has never been proposed. Our experience using this device led us to try this modification of the basic technique so as to further expand the population on which it can be used (3, 4). In cases of the broad or long depressions, two bars are inserted at superior and inferior levels parallel to each other (Figure 5). Here's an overview of some key points in this discussion.

Improved Correction: One of the main reasons for considering a double Pectus Up technique is to achieve better correction of the chest deformity, especially in cases of severe or complex pectus excavatum (5). By using two devices instead of one, surgeons can distribute the force more evenly across the chest wall, potentially leading to a more symmetrical and stable correction.

Increased Stability: Double device placement may provide increased stability during the healing process, particularly in cases where the chest wall is highly flexible or the deformity is extensive. The additional support from the second device can help prevent the sternum from reverting to its original position while the tissues adapt to the new configuration.

Reduced Complications: The use of double bars of Pectus Up may be associated with a lower rate of complications, also compared to the use of two Nuss bars. enhanced stability provided by the second device instead of one, although further research is needed to confirm this potential benefit (6–8).

Challenges and Considerations: While the double Pectus Up technique offers potential advantages, it also presents challenges such as increased technical complexity and the potential for prolonged operative times. Surgeons need to carefully assess each patient's anatomy and the severity of the deformity to determine whether double device placement is appropriate and feasible.

**Figure 6:**
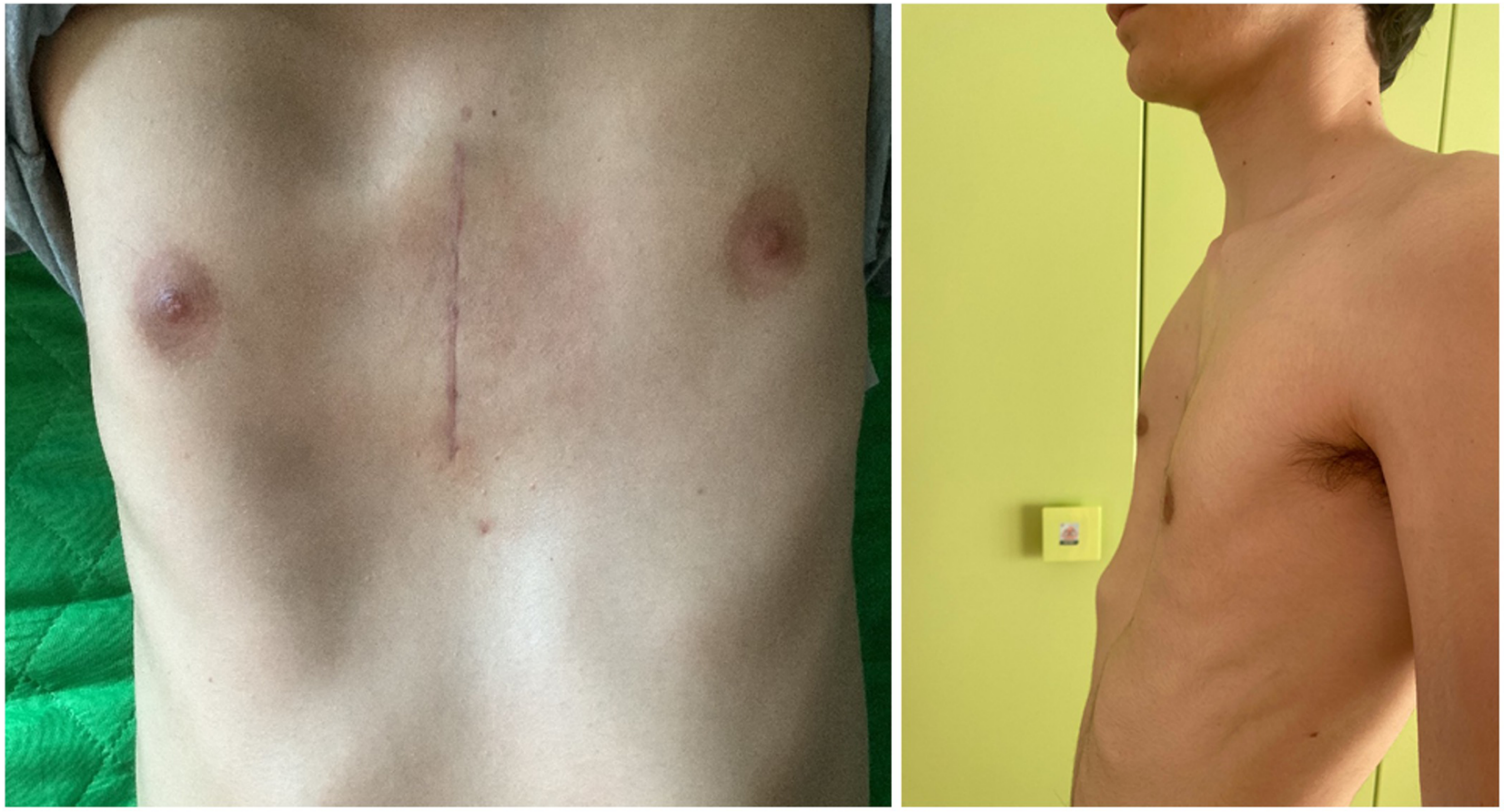
Post-op follow-up at 6 months.

Patient Outcomes: Our outcomes of double Pectus Up procedures have shown promising results in terms of both short-term correction and long-term stability (Figure 6).

In conclusion, the use of a double bar technique in the modified Taulinoplasty procedure is a key area of ongoing clinical research and innovation in the field of pectus excavatum repair. Further studies will certainly be needed, also involving more Institutions that use this technique to validate our initial experience.

## Data Availability

The original contributions presented in the study are included in the article/Supplementary Material, further inquiries can be directed to the corresponding author.

## References

[1] Pardi V Aloi IP Frediani S Martucci C Inserra A. Is chest radiography a valid alternative to computed tomography in evaluation of pectus excavatum? Minerva Pediatr (Torino). (2021). 10.23736/S2724-5276.21.06209-1. [Epub ahead of print] 34128602

[2] Bardají C Cassou L. Taulinoplasty: the traction technique-a new extrathoracic repair for pectus excavatum. Ann Cardiothorac Surg. (2016) 5(5):519–22. 10.21037/acs.2016.09.07 27747186 PMC5056940

[3] Nuss D Kelly RE Jr Croitoru DP Katz ME. A 10-year review of a minimally invasive technique for the correction of pectus excavatum. J Pediatr Surg. (1998) 33(4):545–52. 10.1016/S0022-3468(98)90314-1 9574749

[4] Ravitch MM. The operative treatment of pectus excavatum. Ann Surg. (1949) 129:429–44. 10.1097/00000658-194904000-00002 17859324 PMC1514034

[5] Núñez García B Álvarez García N Aquino-Esperanza J Esteva Miró C Pérez-Gaspar M Jiménez Gómez J Efficacy and safety of taulinoplasty compared with the minimally invasive repair of pectus excavatum approach to correct pectus Excavatum. J Laparoendosc Adv Surg Tech A. (2021) 31(12):1402–7. 10.1089/lap.2021.0216 34847730

[6] Laín A Giralt G Giné C García Martínez L Villaverde I López M. Transesophageal echocardiography during pectus excavatum correction in children: what happens to the heart? J Pediatr Surg. (2021) 56(5):988–94. 10.1016/j.jpedsurg.2020.06.009 32660778

[7] Frediani S Beati F Pardi V Aloi IP Bertocchini A Accinni A Case report: modified taulinoplasty: a new technique for minimally invasive repair of pectus excavatum. Front Surg. (2024) 10:1343515. 10.3389/fsurg.2023.1343515 38283062 PMC10811093

[8] Beati F Frediani S Pardi V Aloi I Bertocchini A Accinni A Case report-every thoracic surgeon’s nightmare: cardiac and lung perforation during placement of nuss bar for pectus excavatum. Front Pediatr. (2023) 11:1241273. 10.3389/fped.2023.1241273 37744443 PMC10513049

